# Supplementary figures and images for: Caution ahead: reassessing the functional morphology of the respiratory organs in amphibious snails
Source: PeerJ. 2021 Sep 20;9:e12161. doi: 10.7717/peerj.12161 (PMC8459726; doi:10.7717/peerj.12161)

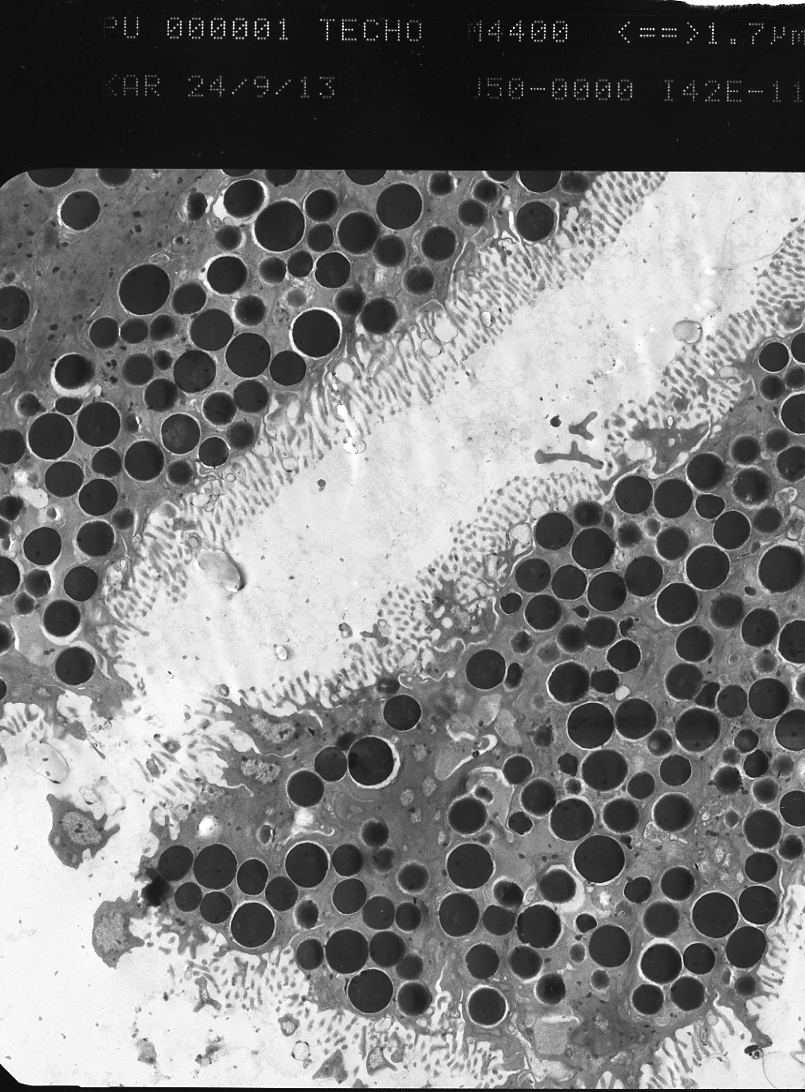

Supplement: Supplemental Information 1 [file peerj-09-12161-s001.zip › Figure 2A.tif]
